# The advancements in targets for ferroptosis in liver diseases

**DOI:** 10.3389/fmed.2023.1084479

**Published:** 2023-03-14

**Authors:** Xiaohong Xiang, Jianbo Gao, Danyang Su, Doudou Shi

**Affiliations:** ^1^Department of Radiology, The First Affiliated Hospital of Zhengzhou University, Zhengzhou, China; ^2^Department of Geriatrics, The Ninth Hospital of Xi'an, Xi'an, Shaanxi, China

**Keywords:** ferroptosis, targets, application, advancement, liver diseases

## Abstract

Ferroptosis is a type of regulated cell death caused by iron overload and lipid peroxidation, and its core is an imbalance of redox reactions. Recent studies showed that ferroptosis played a dual role in liver diseases, that was, as a therapeutic target and a pathogenic factor. Therefore, herein, we summarized the role of ferroptosis in liver diseases, reviewed the part of available targets, such as drugs, small molecules, and nanomaterials, that acted on ferroptosis in liver diseases, and discussed the current challenges and prospects.

## Introduction

Liver disease is a general term for inflammatory and non-inflammatory diseases of the liver, including hepatitis, cirrhosis, steatosis, liver cancer, and others. It is a common and extremely harmful disease that adversely affects human health. Currently, the treatment of end-stage liver disease mainly consists of liver transplantation and symptomatic treatment. Although the quality of life of patients has been greatly improved, the survival rate is still unsatisfactory. Therefore, it is necessary to find more effective treatment strategies for liver diseases.

**Figure 1:**
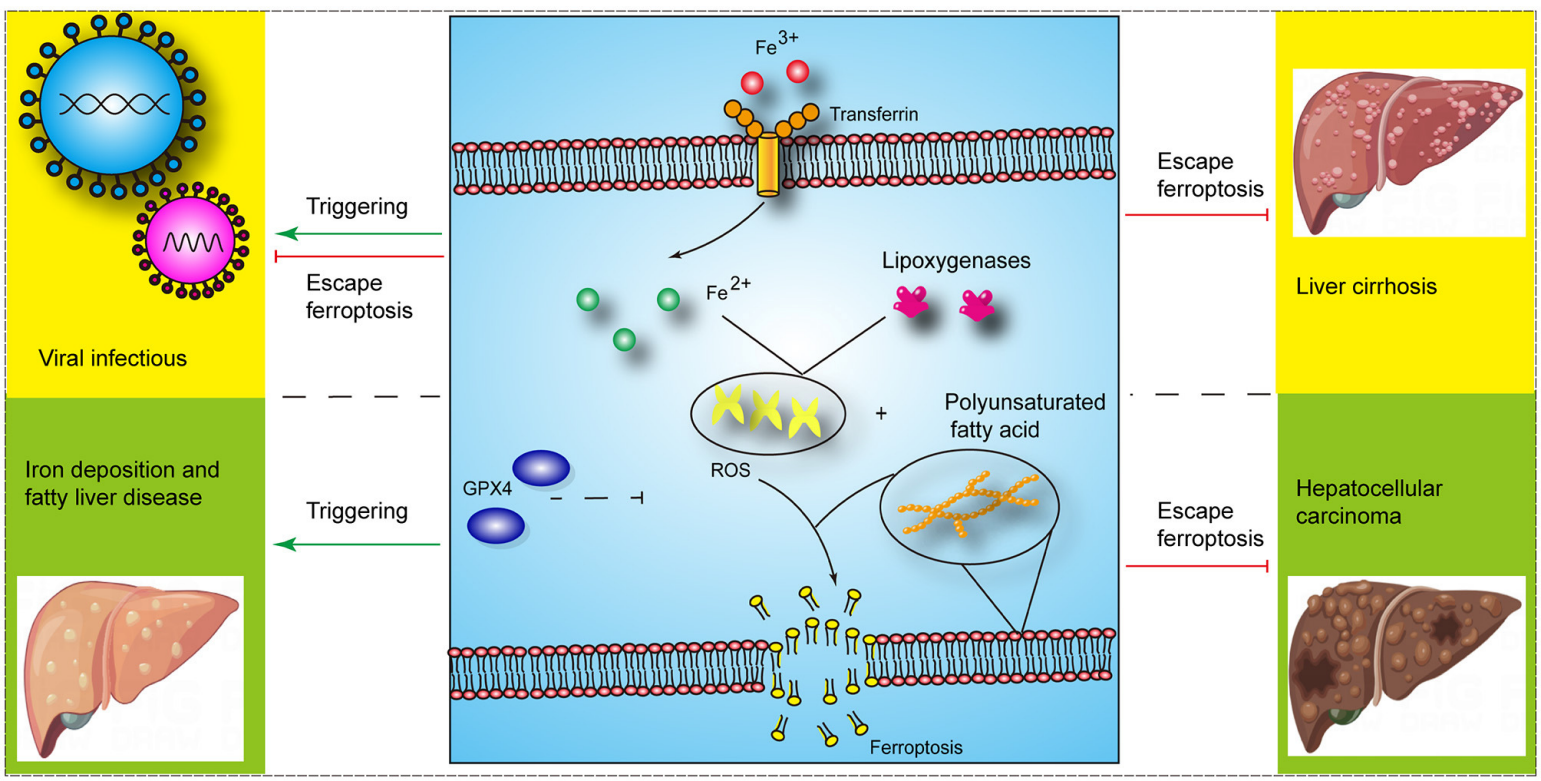
The role of ferroptosis in liver diseases (Some image elements came from Figdraw with permissions).

Ferroptosis is a type of regulated cell death caused by iron overload and lipid peroxidation, which is distinct from pyroptosis, necrosis, and apoptosis. It is involved in various metabolic pathways, including iron, glutathione (GSH), and coenzyme Q metabolism (1). And studies have shown that ferroptosis is associated with the occurrence and development of liver diseases, including cancer, liver fibrosis, and ischemia-reperfusion injury (IRI) (1) (Figure 1). The nuclear factor erythroid 2-related factor 2 (Nrf2)- glutathione peroxidase 4 (GPx4) axis was involved in carbon tetrachloride (CCl4)-mediated acute liver injury in mice (2). Furthermore, the acyl-CoA synthetase long chain family member 4 (ACSL4)-mediated ferroptosis showed a tumor-promoting role in the progression of hepatocellular carcinoma (HCC) from liver injury and a tumor-suppressing role in HCC treatment (3). However, there are still few clinical applications of ferroptosis targets in liver diseases. Therefore, an in-depth understanding of the ferroptosis mechanism will promote the development of therapeutics for ferroptosis in liver diseases. The molecular mechanisms related to ferroptosis have been reviewed by other studies. Thus, herein, we review the role of ferroptosis in liver diseases and summarize the part of available therapeutic targets for ferroptosis in liver diseases.

## The role of ferroptosis in liver diseases

### Ferroptosis and liver injury

Acute liver injury without any underlying liver diseases includes drug-induced liver injury, IRI, and liver injury caused by acute viral infection. Liver injury caused by acetaminophen (APAP) is a common drug-induced liver injury, and IR-induced liver injury mainly includes insufficient perfusion injury of the liver caused by liver transplantation and cardiovascular diseases. A study showed that ferroptosis initiated APAP-induced liver injury, and ferroptosis inhibitors, such as ferrostatin-1 and iron chelator deferoxamine, alleviated APAP-induced liver injury (4). In addition, ferroptosis involved in IRI-induced liver injury, and HECT, UBA and WWE domain containing E3 ubiquitin protein ligase 1 (HUWE1) could antagonize abnormal iron accumulation and ferroptosis to alleviate IRI-induced liver injury (5). Further research showed that ferroptosis-related liver injury employed a variety of regulatory mechanisms, such as exosomes. Mesenchymal stem cells attenuated ferroptosis in acute liver injury by promoting the stability of SLC7A11 via exosomes (6). Moreover, exosome-delivered miR-124-3p and miR-29a-3p from heme oxygenase-1-modified bone marrow mesenchymal stem cells inhibited ferroptosis to alleviate IRI in steatotic grafts (7, 8). Although research on liver injury and ferroptosis has shown promising results, it is mainly at the level of animal models, and there is a long way to go before clinical translation. In addition, studies showed that certain necrosis inhibitors, such as necrostatin-1 (nec1) could also partially inhibit ferroptosis. Therefore, the interaction between ferroptosis and other types of cell death needs to be further explored to better guide ferroptosis treatment strategies. Moreover, inflammatory cells involved in acute liver injury, and ferroptosis have been shown to affect the function of inflammatory cells. For example, the kinase complex mTORC2 promoted longevity of virus-specific memory CD4^+^ T cells by inhibiting ferroptosis (9) and ferroptosis could affect the ability of macrophages to kill intracellular bacteria (10). Thus, whether and how hepatocytes and inflammatory cells ferroptosis affect each other during liver injury requires further research.

Chronic liver injury refers to long-term and chronic damage to liver function caused by multiple factors, and it is mainly seen in chronic viral hepatitis, long-term heavy drinking, and fatty liver. Hepatic stellate cells (HSCs) were rich in iron, which provided the basis for ferroptosis regulation in cirrhosis (11). The study also showed that ferroptosis was closely related to liver cirrhosis. Therefore, elastin and sorafenib treatment could alleviate liver fibrosis in mice by inducing ferroptosis. In addition, sorafenib, a well-known ferroptosis inducer, attenuated liver injury and extracellular matrix accumulation in CCl_4_-induced fibrotic livers by reducing SLC7A11 and GPX4 proteins (12). Ferroptosis affects multiple pathways in liver cirrhosis and is regulated in multiple ways. For instance, ferroptosis could regulate the autophagy signaling pathway in HSCs and dihydroartemisinin attenuated liver fibrosis *via* m6A methylation-involved ferroptosis in HSCs (13). The researchers also investigated ferroptosis in cirrhosis that develops from viral hepatitis. Chen et al. found that chronic hepatitis B virus and/or hepatitis C virus (HCV) infection altered iron metabolism and related genes in the liver (14), and ferroptosis could affect HCV replication and therapeutic efficacy (15). Moreover, hepatitis B virus X protein (HBx) could alleviate the cell death of HSCs by inhibiting ferroptosis, thereby promoting the progression of liver cirrhosis (16). In addition, steatosis and steatohepatitis have been confirmed to be related to ferroptosis, and ferroptosis inhibitors could alleviate liver injury (17), however, the underlying molecular mechanisms are not completely known. Therefore, further research is needed to reveal the role of ferroptosis in steatosis and steatohepatitis. In summary, although several potential ferroptosis targets, such as P53 and ELAVL1, have been identified, the associated studies used only cellular and animal models and the relevant molecular mechanisms are not completely clear. Therefore, further research efforts are needed.

### Ferroptosis and HCC

HCC is one of the important causes of tumor-related deaths, and ferroptosis has been recognized as a tumor suppressor. Therefore, researchers investigated the induction of ferroptosis in HCC cells. Sorafenib is currently used as the first-line drug for the treatment of advanced HCC, and studies have shown that it could induce ferroptosis of HCC cells and thereby inhibit their proliferation (18). And the efficacy of sorafenib was affected by intracellular genetic status; for example, Rb-negative hepatoma cells were more sensitive to sorafenib-induced ferroptosis (19). Additionally, ferroptosis induced by sorafenib could be reversed by ferroptosis inhibitors, such as ferrostatin-1 and activation of p62-Keap1-Nrf2 axis (20, 21). Moreover, sorafenib-induced increase in metallothionein-1G (MT-1G) promoted the resistance of HCC cells to sorafenib (22). This suggests that secondary resistance to sorafenib and gene expression of HCC cells might affect the therapeutic effect of sorafenib; therefore, mechanisms underlying the resistance to sorafenib need to be further elucidated. In addition, ferroptosis-inducing nanoparticles, extracellular lactate levels, and antipsychotic drug haloperidol also participated in inducing ferroptosis of HCC cells (23–25). Taken together, seeking more effective ferroptosis therapeutic targets and strategies is beneficial for HCC.

### The available targets for ferroptosis in liver diseases

The present therapeutic strategies for ferroptosis mainly include genes, RNAs, proteins, small-molecule compounds and nanomaterials. Ferroptosis related nanomaterials consisted of both iron-based and non-iron-based nanomaterials, and they induced ferroptosis by scavenging GSH and inducing the degradation of GPx4 (26). Currently, targets for ferroptosis are mainly divided into ferroptosis inducers and inhibitors. There are four types of ferroptosis-inducing compounds, including those: inhibiting the cysteine (Cys)/glutamate (Glu) reverse exchange (Xc^−^) system, directly or indirectly inactivating GPX4, causing iron overload and activating heme oxygenase 1 (HMOX1) (27). At present, ferroptosis was often regarded as a detrimental factor in certain liver diseases, ferroptosis-inducing agents were mainly used to treat HCC and liver fibrosis (28). Table 1 lists the part of available ferroptosis inducers in liver diseases.

**Table 1:** The part of available ferroptosis inducers and inhibitors in liver disease.

**Effector/reagent**	**Proposed mechanism**	**Liver disease model**	**Reference(s)**
**Ferroptosis inducers**			
Ethyl carbamate	Inhibiting GSH synthesis and suppressing Nrf2 activation	Liver cells	(29)
Celastrol	Targeting peroxiredoxins and HO-1	Liver fibrosis	(30)
Sorafenib	HIF-1α/SLC7A11 pathway	Liver fibrosis; HCC	(12)
Wogonoside	SOCS1/P53/SLC7A11 pathway	Liver fibrosis	(31)
Ellagic acid	Impairing the SNARE complexes formation	Liver fibrosis	(32)
Dihydroartemisinin	Regulating the m6A of BECN1 mRNA	Liver fibrosis	(13)
Acrylamide	Antioxidant imbalance of the XCT-GSH-GPX4 signaling and mitochondrial dysfunction	Liver fibrosis	(33)
BECN1	xCT/GPX4 axis	Liver fibrosis	(34)
HBV X protein (HBx)	EZH2 mediated SLC7A11 suppression	Acute liver failure	(35)
TRIM26	SLC7A11 Ubiquitination	Liver fibrosis	(36)
Celastrol	Targeting peroxiredoxins and HO-1	Liver fibrosis	(30)
Corosolic acid	Upregulating HERPUD1	HCC	(37)
PCDHB14	Downregulating the expression of SLC7A11	HCC	(38)
Lenvatinib	Fibroblast growth factor receptor-4 inhibition	HCC	(39)
A multifunctional vanadium-iron-oxide nanoparticle	Increasing reactive oxygen species (ROS)	HCC	(40)
COMMD10	Inhibits HIF1α/ ceruloplasmin (CP) loop	HCC	(41)
Ketamine	lncRNA PVT1/miR-214-3p/GPX4	HCC	(42)
Dihydroartemisinin	Upregulation of CHAC1 expression	HCC	(43)
	Promoting the formation of PEBP1/15-LO		(44)
O-GlcNAcylation	YAP/TFRC pathway	HCC	(45)
Quiescin sulfhydryl oxidase 1	Driving EGFR endosomal trafficking and inhibiting NRF2 activation	HCC	(46)
Acid-degradable tumor targeted nanosheets Cu-Hemin-PEG-Lactose acid (Cu-Hemin-PEG-LA)	Consuming intracellular glutathione (GSH) content and increasing the expression of heme oxygenase 1 (HMOX1) protein	HCC	(47)
iRGD with sorafenib-loaded iron-based metal-organic framework (MIL-101(Fe)@sor NPs)	Decreasing GPX-4 expression level	HCC	(48)
Cascaded copper-based metal-organic framework (MOF)	Modulating glutathione and cyclooxygenase-2	HCC	(49)
Erastin	NEAT1/miR-362-3p/MIOX axis	HCC	(50)
RSL3	GPX4	HCC	(51)
Aspirin	Restricting NF-κB p65-activated SLC7A11 transcription	HCC	(52)
Auranofin	TXNRD1	HCC	(53)
**Ferroptosis inhibitors**			
miR-23a-3p	ETS1/miR-23a-3p/ACSL4	HCC	(21)
C8orf76	Up-regulating SLC7A11	HCC	(54)
miR-124-3p	Inhibiting prostate six transmembrane epithelial antigen 3 (STEAP3)	Schemia-reperfusion injury in steatotic grafts	(8)
miR-29a-3p	Downregulating the expression of Ireb2	Steatotic liver ischemia-reperfusion injury	(7)
miR-222	Downregulating the expression of TFRC	Liver fibrosis	(55)
Bicyclo	Nrf2-GPx4 axis	Acute liver injury	(2)
Maresin1	Nrf2/HO-1/GPX4	Acute liver injury	(56)
Fucoidan	Decreased divalent metal transporter 1 (DMT1) and ferroportin1 (FPN1) expression	Liver injury in rats exposed to alcohol	(57)
MCTR1	Promoting NRF2 expression	Hepatic ischemia-reperfusion injury	(58)
Astaxanthin	Nrf2/HO-1 Pathway	Acetaminophen-induced liver injury	(59)
3,4-dihydroxyphenylethyl alcohol glycoside	Inhibiting the expression of ERK, HO-1, NLRP3, Caspase1 (p20) and Gasdermin-D and upregulating the expression of GPX4	Acetaminophen-induced acute liver failure in mice	(60)
PPARα	Directly inducing Gpx4 expression by binding to a PPRE element within intron 3	Iron overload in mouse liver	(61)
Fibroblast growth factor 21	Inducing HO-1 inhibition and NRF2 activation	Liver injury and fibrosis	(62)
Dehydroabietic acid	Keap1/Nrf2-ARE signaling pathway	Non-alcoholic fatty liver disease	(63)
Niujiaodihuang detoxify decoction	Enhancing glutathione synthesis	Acute liver failure	(64)
G6PD	Targeting cytochrome P450 oxidoreductase	HCC	(65)
α-Enolase 1 (ENO1)	Degrading the mRNA of iron regulatory protein 1	HCC	(66)
AdipoR1	Nrf2/xCT Pathway	HCC	(67)
ZNF498	Attenuating the p53 Ser46 phosphorylation	HCC	(68)
ABCC5	Stabilizing SLC7A11 protein	HCC	(69)
Rosigliazone	ACSL4	Hepatitis; liver Injury; NAFLD; NASH	(70–74)
α-tocopherol	Oxidation		
Deferoxamine	Free iron		
Debenone, CoQ10	CoQ10		
Selenium	Selenoproteins		
Fer-1	Free radical	Hemochromatosis, thalassemia; NAFLD; NASH	(75–78)
MitoTEMPO			
Enoyl coenzyme A hydratase 1	Erk signaling pathway	Non-alcoholic steatohepatitis	(79)
Thymosin beta 4	Up-regulating GPX4	Non-alcoholic fatty liver	(80)

Ferroptosis inhibitors work by reducing iron overload and peroxidation levels and scavenging peroxidation products (81). Of these, the prominent ones are iron chelators that reduce iron overloads, such as deferoxamine and deferiprone. A small number of iron chelators are used in the clinic or clinical trials. And the library of novel deferoxamine compounds is created and updated regularly (40). Table 1 lists the part of available ferroptosis inhibitors in liver diseases. Furthermore, nec1 inhibited necrosis and ferroptosis in primary renal tubular and mouse heart transplantation models (82), but its use in liver disease is not reported. So far, the role of ferroptosis inhibitors in liver diseases has mainly been reported in animal models, and there are only a few reports about its clinical application. Therefore, additional research is needed to provide evidence for targeted ferroptosis in liver diseases. And although nanomaterials targeting ferroptosis have improved their targeting properties through special modifications such as biofilm and PUFA modification (83), their safety for *in vivo* application has not been effectively demonstrated.

## Discussion

Ferroptosis plays an important role in the occurrence, development, and treatment of liver diseases. Researchers have screened and identified several ferroptosis inducers and inhibitors using animal experiments and small molecule compound libraries. However, evidence regarding the distribution, metabolism, excretion and use of these targets is lacking. Secondly, the role of these targets in the progression of chronic liver disease to HCC may be variable, and the underlying mechanisms are unknown. Additionally, how can targets be adjusted at different stages of liver diseases is not clear. Thirdly, the targeting of these agents needs to be improved. Studies have shown that these targets promote or inhibit ferroptosis of inflammation cells and thus affect their immune function. At present, there is little relevant evidence and the underlying mechanism needs to be elucidated. Finally, current nanomedicine technologies enable highly specific targeting of these agents, and nanomaterial-related ferroptosis promoters and inhibitors have been studied in cancers. There is a long way to go before we can effectively target ferroptosis to treat liver diseases, but we believe that combining efforts from over the world will help us realize the therapeutic strategy.

## Author contributions

XX and JG designed the study. DSu, XX, and DSh revised the manuscript. All authors contributed to the article and approved the submitted version.
